# Feeding the Outer Bran Fraction of Rice Alters Hepatic Carbohydrate Metabolism in Rats

**DOI:** 10.3390/nu12020430

**Published:** 2020-02-07

**Authors:** Mana Kawaguchi, Nao Nishikoba, Saki Shimamoto, Shozo Tomonaga, Rukana Kohrogi, Yoko Yamauchi, Yoshikazu Fujita, Akira Ohtsuka, Daichi Ijiri

**Affiliations:** 1 Department of Biochemical Science and Technology, Kagoshima University, 1-21-24 Korimoto, Kagoshima 890-0065, Japan; k4475433@kadai.jp (M.K.); k9672974@kadai.jp (N.N.); k8429056@kadai.jp (S.S.); k1158178@kadai.jp (R.K.); ohtsuka@chem.agri.kagoshima-u.ac.jp (A.O.); 2 Division of Applied Biosciences, Graduate School of Agriculture, Kyoto University, Kitashirakawa Oiwake-cho, Sakyo-ku, Kyoto 606-8502, Japan; shozo@kais.kyoto-u.ac.jp; 3 Shokkyo Co., Ltd., 5-9 Matsukawacho, Minami-ku, Hiroshima 732-0826, Japan; yamauchi@shokkyo.co.jp (Y.Y.); fujita-y@shokkyo.co.jp (Y.F.)

**Keywords:** metabolomics, rice bran, short-chain fatty acid, fatty acid synthesis, gluconeogenesis

## Abstract

Dietary intake of fiber-rich food has been reported to contribute to multiple health benefits. The aim of the current study is to investigate the effects of a diet containing the outer bran fraction of rice (OBFR), which is rich in insoluble fiber, on the intestinal environment and metabolite profiles of rats. Fourteen 8-week-old male Sprague–Dawley rats were divided into a control group and an OBFR group. For a period of 21 days, the control group was fed a control diet, while the OBFR group was fed a diet containing 5% OBFR. Metabolomics analysis revealed drastic changes in the cecal metabolites of the rats fed the OBFR diet. Furthermore, in the plasma and liver tissue, the concentrations of metabolites involved in pyruvate metabolism, the pentose phosphate pathway, gluconeogenesis, or valine, leucine, isoleucine degradation were changed. Concordantly, the OBFR diet increased the expression of genes encoding enzymes involved in these metabolic pathways in the livers of the rats. Collectively, these results suggest that the OBFR diet altered the concentrations of metabolites in the cecal contents, plasma, and liver, and the hepatic gene expressions of rats, and that this may have mainly contributed to carbohydrate metabolism in the liver.

## 1. Introduction

Dietary fiber can be classified as insoluble (cellulose, hemicellulose, and lignin) or soluble (pectin and gum), depending on its solubility in water. It has been well established that dietary fiber contributes to multiple health benefits [1] by reducing caloric absorption [2,3] and increasing the fecal excretion of energy, protein, and fat [1,4]. Interestingly, ingesting insoluble fiber has been found to reduce energy digestibility, body weight gain, or blood lipid levels in animals, e.g., feeding a dietary fiber-rich by-product of the soy milk industry to healthy rats for 4 weeks showed decreases in weight gain and serum total cholesterol level [5]. In addition, we previously found that feeding the outer bran fractions of rice (OBFR) to rats for 3 weeks enhanced fecal lipid excretion, and consequently reduced abdominal lipid accumulation [6].

Rice bran is a by-product of the rice milling process, and consists of several cellular layers, including the pericarp, tegmen, and aleurone, and is thus rich in fiber [7]. Eating rice bran can substantially improve bowel movement and fecal excretion [8], and reduce plasma triacylglycerol and total cholesterol levels in rats [9]. Feeding rats rice bran oil also increased fecal weight and fecal bile acid excretion [10], and eating rice bran significantly improved bowel movement in humans [11], although the effects of dietary rice bran on plasma lipid profiles were reportedly small [12]. Rice bran contains functional components including ferulic acid and γ-oryzanol, which contribute to the inhibition of cholesterol absorption in the intestines and its excretion via the feces [13,14]. In addition, phytic acid reportedly inhibited the activity of porcine pancreatic lipase [15], and this inhibition reduced the availability of dietary fat in the intestine [16].

Multi-break milling systems have recently become prevalent in the rice industry. In such systems, multiple milling machines are used to remove more bran from the rice kernels after the initial break. The outer layer of rice bran mainly consists of the pericarp, which is richer in lignin than the inner layer, while the amounts of pectic substances, hemicellulose, and α-cellulose are approximately equal in the inner and outer layers [17]. In a previous study, we collected the OBFR from the first and second breaks of a commercial quadruple-break milling system and found that the OBFR contains γ-oryzanol and phytic acid at 1.2-fold higher abundance than whole rice bran [6]. The aim of the current study is to examine the effects of dietary OBFR on the intestinal environment and plasma, hepatic, and cecal metabolite profiles of rats.

## 2. Materials and Methods

### 2.1. Diet Preparation

A control diet (AIN-93G) and a diet containing 5% OBFR were prepared in advance for use in the study. OBFR was collected as previously described [6]. The crude protein (CP), neutral detergent fiber (NDF), and gross energy (GE) components of the two diets were designed to be equal. The ingredient compositions and analyzed contents of the two diets are shown in Table 1.

### 2.2. Animals and Treatments

Fourteen 7-week-old male Sprague–Dawley rats (weighing approximately 200 g upon arrival to the animal facility) were purchased from Japan SLC Co. Ltd. (Shizuoka, Japan). This strain of rats was chosen as it is commonly used for animal studies on the effects of dietary fibers on hepatic metabolism [18]. The study was conducted in accordance with the guidelines of the Animal Care and Use Committee of Kagoshima University (approval number A16011, issued on 8 June 2016). All rats in the study were housed individually in wire-bottomed aluminium cages (152 × 201 × 170 mm) and initially provided water and the control diet ad libitum for 6 days in a temperature-controlled room at 25 °C. The rats were checked daily for any health or welfare problems. No signs of pain, suffering or distress were observed before or during the study.

At 8 weeks of age, the rats were randomly divided into two groups: one that was fed the control diet and another that was fed the OBFR diet. Every rat’s body weight was measured once per week, and every rat’s food intake was measured once per day. Fecal samples were collected during the last 3 days of the experiment. All rats were killed via cervical dislocation under carbon dioxide-induced anesthesia, then the heart, liver, kidney, caecum, and abdominal fat were collected and weighed. Cecal content was transferred into a plastic test tube and stored at −80 °C prior to analysis. A portion of the cecal content was weighed in another plastic test tube and diluted 10-fold in double-distilled water, then the pH of the suspension was measured using a pH meter (HM-30G, DKK-TOA Corporation, Tokyo, Japan). Blood samples were collected in heparinized test tubes, which were promptly centrifuged at 5900× *g* for 10 min at 4 °C to separate plasma, which were then stored at −30 °C prior to analysis. The levels of glucose in the plasma were measured using a Fuji DRI-CHEM 3500 analyzer (Fujifilm, Tokyo, Japan), in accordance with the manufacturer’s instructions.

### 2.3. Dietary Digestibility

The dietary and fecal amounts of CP, ether extract (EE), crude fiber, crude ash, nitrogen-free extract (NFE), and NDF were measured in accordance with AOAC procedures.

### 2.4. Determination of Organic acid Content in Cecal Content

The amounts of specific organic acids present in cecal content were measured using a high-performance liquid chromatography system (Jasco, Tokyo, Japan), in accordance with the method described by Miwa et al. [19]. The organic acids measured were lactic acid, acetic acid, propionic acid, butyric acid, isobutyric acid, isovaleric acid, and valeric acid. The UV–vis detector (UV-2075 PLUS, Jasco) was set to 400 nm, and a YMC-Pack FA column (6 × 250 mm; YMC Co., Ltd., Kyoto, Japan) was used with a column oven (CO-2065 PLUS) heated to 50 °C. The mobile phase consisted of acetonitrile–methanol–water (30:16:54 *v*/*v*, pH 4.5), and the flow rate was 1.2 mL/min. The organic acids were labeled using a short and long-chain fatty acid analysis kit (YMC, Kyoto, Japan) in accordance with the manufacturer’s instructions.

### 2.5. Sample Preparation for Gas Chromatography/Mass Spectrometry Analysis

Metabolomic analysis was performed using gas chromatography/mass spectrometry (GC/MS) as previously described [20], with some modifications. Frozen liver samples were pulverized into a powder using a crusher (T- 351, Tokyo Unicom, Tokyo, Japan) with frozen carbon dioxide. Approximately 20 mg of the freeze-fractured liver samples and cecal content samples and 50 μL of plasma were then suspended in 250 µL of methanol–chloroform–water (5:2:2) and 5 µL of 1 mg/mL 2-isopropylmalic acid as an internal standard. Samples were then mixed in a shaker at 1200 rpm at 37 °C for 30 min, then centrifuged at 16,000× *g* at 4 °C for 5 min. Next, 225 µL of the supernatant was mixed with 200 µL of distilled water and vortex-mixed, followed by centrifugation at 16,000× *g* at 4 °C for 5 min. Subsequently, 250 µL of the supernatant was dried under a vacuum using a centrifugal evaporator (RD-400; Yamato Scientific, Tokyo, Japan) after cooling at −80 °C for 10 min. Methoxyamine hydrochloride in pyridine (20 mg/mL, 40 µL) was then added to the tubes and they were vortex-mixed, then shaken at 1200× *g* at 30 °C for 90 min in the dark for oximation. N-methyl-N-trimethylsilyltrifluoroacetamine (20 µL) was then added to each tube and the contents were vortex-mixed. To prepare trimethylsilyl derivatives, the tubes were shaken at 1200× *g* at 37 °C for 45 min in the dark.

### 2.6. GC/MS Analysis

GC/MS analysis was performed as previously described [21], using a GC/MS-QP2010Ultra (Shimadzu Corporation, Kyoto, Japan). A 30-m × 0.25-mm i.d. DF: 0.25-mm InertCap 5MS/NP (GL-Science, Tokyo, Japan) was used as the GC column. The inlet temperature was 230 °C, and the column flow rate was 1.12 mL/min. Helium was used as the carrier gas. The column temperature was maintained at 80 °C for 2 min, then increased to 320 °C at a rate of 15 °C/min and held for 6 min. The transfer line and source temperatures were 250 and 200 °C, respectively. Electron ionization was performed at 70 V. Twenty scans per second were recorded over a mass range of 85–500 *m*/*z*. In addition, an injection of a standard alkane mixture (C9-C40) was run through the column prior to samples. The retention time data of each peak in the mixture were then used as a reference for tentative identification.

### 2.7. Data Processing

The GC/MS analysis data were exported in net CDF format, then converted to ABF format, and peak detection and alignment were performed using MS-DIAL version 3.08 [22] under the conditions shown in Table S1. Raw peak exaction, baseline filtering and calibration of the baseline data, peak alignment, deconvolution analysis, peak identification, and integration of peak height were performed. To minimize the number of missing values, peaks with a similarity of >70% and a retention index within ±10% were accepted via comparison with the compound database (GL-Science DB InertCap 5MS-NP, Kovats RI, 494 records) available from PRIMe (http://prime.psc.riken.jp/). Metabolite levels were semi-quantified using the peak area of each metabolite relative to the internal standard (2-isopropylmalic acid). The level of each metabolite in the control cells was set to 100. Additionally, the levels of each metabolite are shown as ratios derived via comparison with the control group of rats.

### 2.8. RNA Extraction and Quantitative Real-Time PCR

Rat livers were homogenized in ISOGENII (Nippon gene, Tokyo, Japan) in accordance with the manufacturer’s instructions. Quantitative real-time PCR was performed as previously described, with minor modifications [23]. In brief, complementary DNA was synthesized from 60 ng of RNA per 10 µL of reaction solution using the PrimeScript RT Reagent Kit (PR036A; Takara, Shiga, Japan). Samples were incubated at 37 °C for 15 min, 85 °C for 5 s, and 4 °C for 5 min. Gene expression levels were measured by real-time PCR using the 7300 Real-Time PCR system (Applied Biosystems, Foster City, CA, USA) with SYBR Select Master Mix (Applied Biosystems). Thermal cycling conditions were initial incubation at 50 °C for 2 min, 95 °C for 2 min, then 45 cycles of 95 °C for 15 s, 55 °C for 15 s, and 72 °C for 1 min. The primers used in the study are listed in Table S2. Because there were no significant differences in the RPS18 cycle threshold values of each group, the level of RPS18 was used as an internal standard. Gene expression levels are shown as ratios derived via comparison with the control group of rats.

### 2.9. Statistical Analysis

Student’s *t*-test was used to compare the means of the two groups, and all data are presented as mean ± the standard error of the mean. This analysis was performed using R [24]. Statistical significance was set at *p* < 0.05 for all comparisons.

To explore the metabolic pathways affected, quantitative enrichment analysis by pathway-associated metabolite sets of MetaboAnalyst 4.0 [25], an established form of metabolite set enrichment analyses, was performed using either plasma or liver metabolite concentrations obtained via non-targeted analysis. Differences were considered significant at *p* < 0.05.

## 3. Results

### 3.1. The Effects of Feeding OBFR on Growth Performance and Feed Digestibility

Throughout the 21 days of the feeding period, neither the mean body weight nor the mean body weight gain differed significantly between the two groups (Table 2). In addition, the OBFR diet had no significant effect on the weight of the individual organs or tissue types (i.e., liver, heart, kidney, abdominal fat, and soleus muscle) at the end of the 21-day feeding period (Table 2). Although the OBFR diet had no significant effect on the feed intake, it significantly reduced the feed efficiency of the rats, compared with their counterparts fed the control diet (*p* < 0.05). Concordantly, the OBFR diet significantly reduced the digestibilities of CP, EE, and NFE (Table S3).

There were no significant differences in the measured blood parameters (glucose, triacylglycerol, and total cholesterol) between the treatment groups (Table 2). In addition, the plasma 3-methylhistidine (3-MeHis) concentration did not differ between the two groups (Table 2).

### 3.2. The Effects of Feeding OBFR on pH and Organic Acid Concentration of Cecal Contents

The OBFR diet significantly reduced the pH of the rats’ cecal contents (7.44 ± 0.06) compared with their control diet counterparts (8.19 ± 0.04). And, feeding the OBFR diet significantly increased the concentrations of acetic acid, butyric acid, and propionic acid, while it did not affect the concentrations of isobutyric acid, isovaleric acid, and, valeric acid (Table 3).

### 3.3. Untargeted GC/MS-Based Metabolomics Analysis in Cecal Contents, Plasma, and Liver

One thousand six hundred and twenty features were detected and a total of 64 metabolites were identified in the rats’ cecal contents. Of these 64 metabolites, 10 were significantly increased by feeding the OBFR diet, and 12 were decreased (Table 4). In the plasma, 382 features were detected and 54 metabolites were identified, of which 10 were significantly increased by feeding the OBFR diet, and three were decreased (Table 5). In the liver, 751 features were detected and 73 metabolites were identified, of which 10 were significantly increased by feeding the OBFR diet, and 4 were decreased (Table 6).

**Figure 1:**
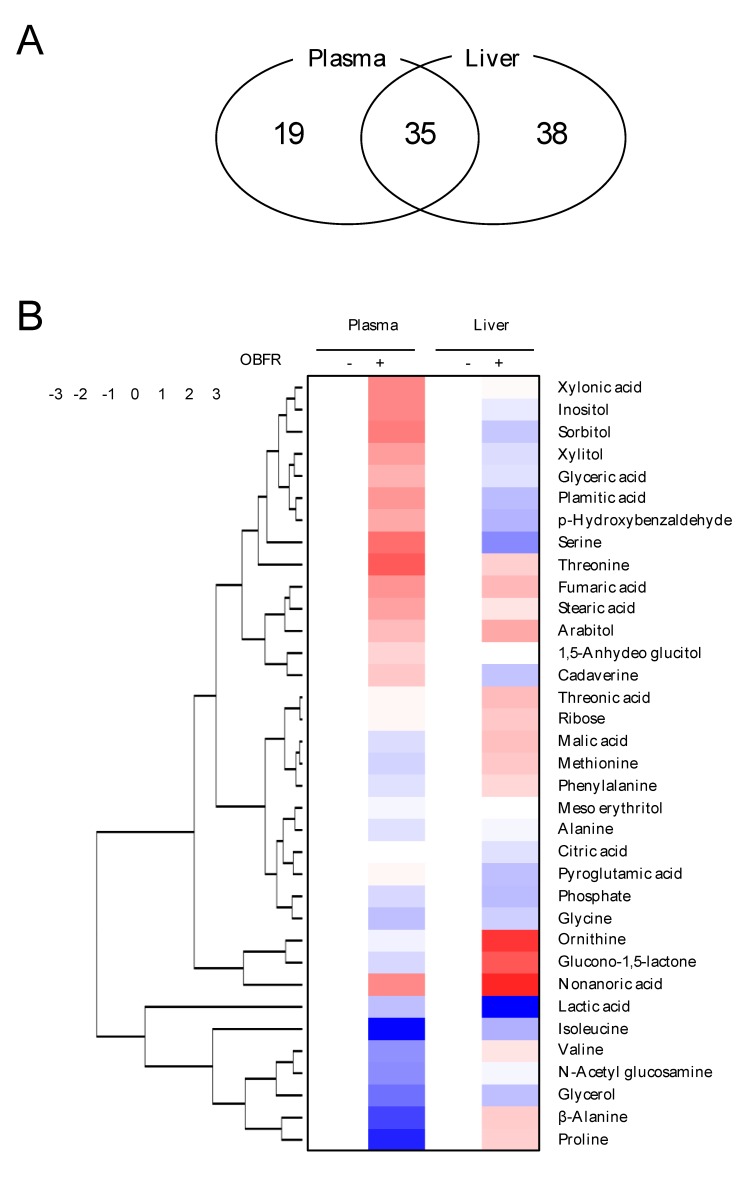

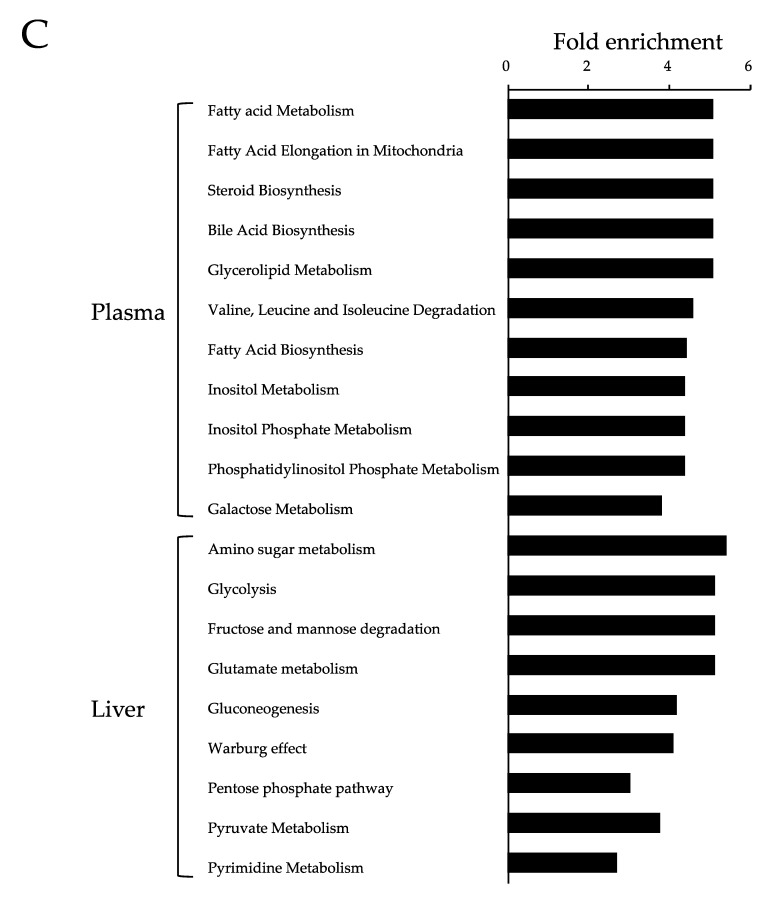
Global changes in metabolite abundance in the plasma and livers of rats fed the OBFR diet. (**A**) The number of metabolites in the plasma and liver. (**B**) The relative abundance of 35 metabolites that were detected in both the plasma and liver, shown as a heat map. The content of each metabolite is shown as a log_2_ value, with the log_2_ value of the control group set to zero. (**C**) The metabolic pathways significantly affected by feeding OBFR, as determined through the enrichment analysis of metabolites which differed significantly in abundance between the groups. OBFR, outer bran fraction of rice.

A total of 35 metabolites were detected in both the plasma and liver tissue, while 19 and 38 metabolites were detected only in the plasma and liver, respectively (Figure 1A). Figure 1B shows a hierarchical analysis performed using the relative abundance of these metabolites detected in both the plasma and liver. The enrichment analysis indicated that feeding OBFR affected 11 and 9 metabolic pathways in the plasma and liver, respectively (Figure 1C).

### 3.4. The Effects of Feeding OBFR on Gene Expressions Encoding Metabolic Enzymes

**Figure 2:**
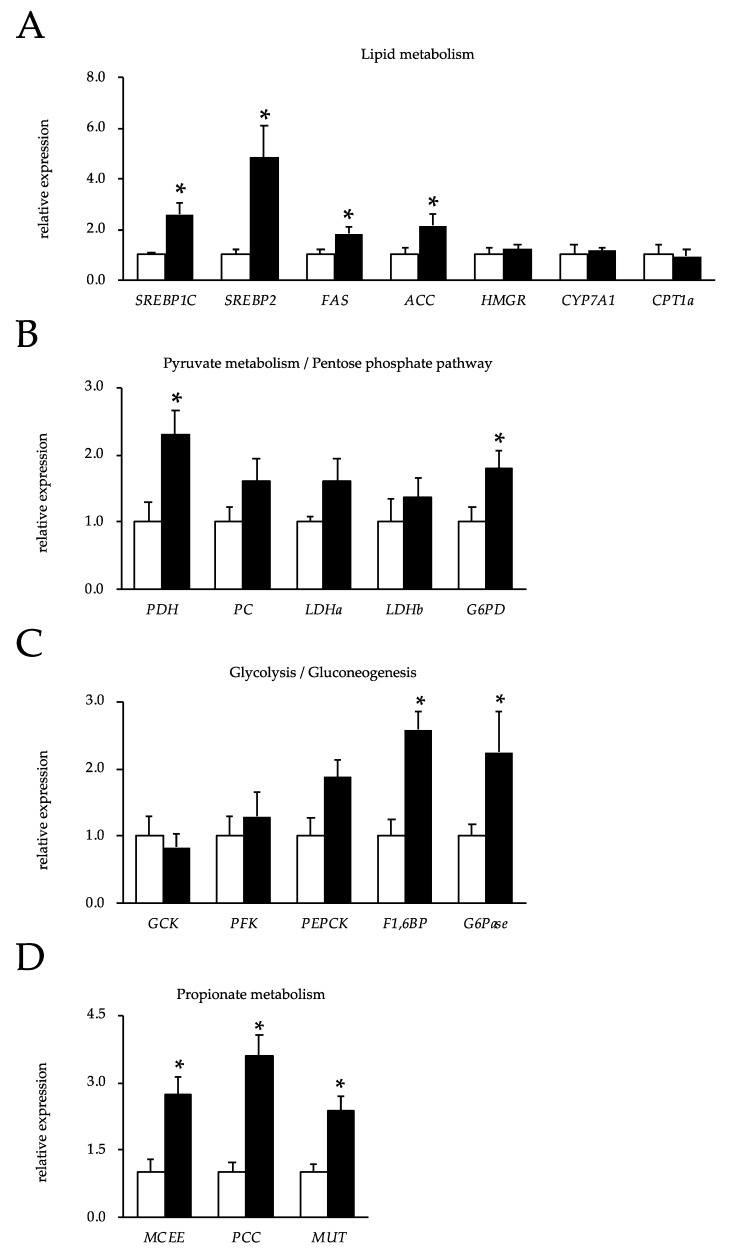

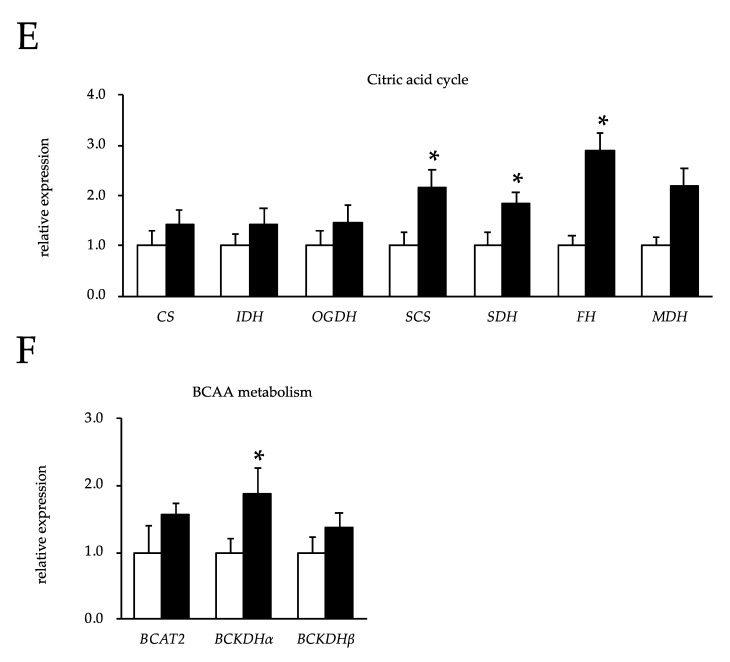
Gene expression analysis of livers from rats fed the OBFR diet. The expression levels of genes encoding enzymes involved in (**A**) lipid metabolism, (**B**) pyruvate metabolism and the pentose phosphate pathway, (**C**) glycolysis and gluconeogenesis, (**D**) propionate metabolism, (**E**) the citric acid cycle, and (**F**) BCAA metabolism. The open bars show the averages of the rats fed the control diet, while the closed bars show the averages of the rats fed the OBFR diet. Values are expressed as means ± the standard error (*n* = 7). * OBFR differs significantly from the control (*p* < 0.05). OBFR, outer bran fraction of rice; SREBP, sterol regulatory element-binding protein; FAS, fatty acid synthase; ACC, acetyl-CoA carboxylase; HMGR, hydroxymethylglutaryl-CoA reductase; CYP7A, cholesterol 7 alpha-hydroxylase; CPT, carnitine/palmitoyl-transferase; PDH, pyruvate dehydrogenase complex, component X; PC, pyruvate carboxylase; LDH, lactate dehydrogenase A; G6PD, glucose-6-phosphate dehydrogenase; GCK, glucokinase; PFK, phosphofructokinase; PEPCK, phosphoenolpyruvate carboxykinase; F1,6BP, fructose 1,6-bisphosphatase; G6Pase, glucose-6-phaosphatase; MCEE, methylmalonyl CoA epimerase; PCC, propionyl-CoA carboxylase; MUT, methylmalonyl CoA mutase; CS, citrate synthase; IDH, isocitrate dehydrogenase; OGDH, oxoglutarate dehydrogenase; SCS, succinyl-CoA synthetase; SDH, succinate dehydrogenase; FH, fumarate hydratase; MDH, malate dehydrogenase; BCAT, branched-chain aminotransferase; BCKDH, branched-chain α-ketoacid dehydrogenase complex alpha.

Because the liver plays a major role in metabolism and has a number of functions, we explored the expression levels of genes encoding enzymes related to metabolic processes. Figure 2A shows the expression levels of genes encoding enzymes involved in lipid metabolism in the livers of rats. The OBFR diet increased the mRNA expression of SREBP1C, SREBP2, FAS, and ACC. On the other hand, it did not affect gene expressions encoding HMGR, CYP7A1, and CPT1a mRNA. Feeding the OBFR diet increased the mRNA expression of PDH and G6PD, but did not affect the mRNA expressions of PC, LDHa, or LDHb (Figure 2B).

In addition, feeding the OBFR diet did not affect the expression levels of the genes encoding GCK and PFK, whereas it significantly increased the expression levels of the genes encoding PEPCK mRNA and F1,6BP mRNA (Figure 2C). Although there was no significant difference between these groups, G6Pase mRNA expression tended to be higher in the livers of the rats fed the OBFR diet. 

Furthermore, feeding the OBFR diet increased the expressions of the genes encoding MCEE, PCC, and MUT in the livers of the rats (Figure 2D). Although feeding the OBFR diet did not change the mRNA expressions of CS, IDH, and OGDH, it significantly increased the mRNA expressions of SCS, SDH, FH, and MDH (Figure 2E). In addition, feeding the OBFR diet did not change the BCAT2 mRNA expression level in the liver, while it significantly increased the BCKDCα mRNA level (Figure 2F).

## 4. Discussion

Feeding the OBFR diet affected neither mean body weight nor mean body weight gain. In addition, it had no significant effect on the weight of the individual organs or tissue types (i.e., heart, liver, kidney, abdominal fat, and soleus muscle). However, although the OBFR diet had no significant effect on feed intake, it significantly reduced the feed efficiency and the digestibilities of CP, EE, and NFE of the rats, compared with their counterparts fed the control diet. The decreases in the feed efficiency and digestibility may have been due to the high dietary fiber content of the OBFR. It has been well established that eating dietary fiber reduces the digestibility of ingested nutrients by disrupting nutrient absorption [2,3]. More specifically, feeding insoluble fiber to rats reduced the digestibility of CP and EE [4,5,26]. Given that OBFR is rich in insoluble fiber (i.e., lignin, hemicellulose and pectic substance) [16], these results suggest that the OBFR diet reduced feed efficiency by reducing the digestibility of CP, EE, and NFE.

However, as was found for the body weight, body weight gain, and individual organ and tissue weights, there were no significant differences in the measured blood parameters (glucose, triacylglycerol, and total cholesterol) between the treatment groups. In addition, the plasma 3-MeHis concentration, which serves as an index for muscle protein degradation [27], did not differ between the two groups, indicating that feeding the OBFR diet did not affect the muscle protein degradation rate. However, the digestibility of the NDF in the rats fed the OBFR diet was double that of their control diet counterparts. These findings suggest that the OBFR diet may have been fermented and used in the rats’ intestines.

Since the OBFR diet significantly reduced the pH of the rats’ cecal contents, we performed untargeted GC/MS-based metabolomics analysis to evaluate the impact of feeding the OBFR diet on the cecal metabolites. A total of 64 metabolites were detected in the rats’ cecal contents. The principal component analysis score scatter-plot based on all the identified metabolites in the cecal contents (Figure S1A) suggested that the cecal metabolites were distinctly distinguishable between these two groups. In addition, feeding the OBFR diet markedly increased the concentrations of some organic compounds with lower pKa (i.e., 3-hydroxyphenylacetic acid, adenine, glutaric acid, glyceric acid, and 3-hydroxybenzoic acid), suggesting that these compounds may have contributed to the lower pH of the cecal contents of the rats fed the OBFR diet.

Dietary fiber in the intestinal tract is converted into short-chain fatty acids (SCFAs) by some species of enteric bacteria, such as *Lactobacillus*, *Streptococcus* and *Bifidobacterium*, and eating a fiber-enriched diet has reportedly resulted in a lower pH in the intestinal tract in humans [28]. We therefore determined the organic acid concentrations involving SCFA in the intestinal contents, and found that feeding the OBFR diet markedly increased the concentrations of acetic acid, butyric acid, and propionic acid. This is in agreement with the earlier studies showed that feeding diet containing rice bran markedly increases in intestinal SCFA in rats [29,30]. Furthermore, 95% of the SCFAs produced in the cecum and large intestine are rapidly absorbed by colonocytes [31,32,33,34], and SCFAs have reportedly been found in hepatic, portal, and peripheral blood [35,36]. It has therefore been suggested that SCFAs provide 10% of the daily caloric requirements in humans [37]. In this study, the concentrations of acetic acid, butyric acid, and propionic acid in the cecal content were 1.5–2.8-fold higher in the rats in the OBFR group, and this may have affected the metablites profiles of these rats.

Untargeted GC/MS-based metabolomics analysis was therefore performed to investigate the effects of the OBFR diet on the metabolites profiles of the plasma and liver of the rats. Although 35 metabolites were detected in both the plasma and liver tissue, 19 and 38 metabolites were detected only in the plasma and liver, respectively. Because the liver plays a major role in metabolism and has a number of functions, a relatively large number of metabolites were confirmed in the liver compared to the plasma under the same condition for detection. On the other hand, since metabolites in plasma reflect the state of the rat’s whole body, the 19 metabolites detected only in the plasma might be derived from tissues except for the liver. As was found for the cecal content, the principal component analysis score scatter plots for the plasma and liver (Figure S2B,C, respectively) suggested that the two treatment groups were distinctly distinguishable.

In this study, 64, 54, and 73 metabolites were identified in the rats’ cecal contents, plasma, and liver, respectively. The numbers of identified metabolites were relatively less than other studies using an untargeted metabolomics approach, e.g., 140, 167, and 207 metabolites were identified in the rat’s plasma [38], liver [39], and feces [40], respectively. The reason for this might be due to the condition for metabolite identification shown in Table S1. In the GC/MS untargeted metabolomics of this study, the annotation reached level 2 on the scale of confidence in metabolite identification, as defined by the Chemical Analysis Working Group of the Metabolomics Standards Initiative [41]. Indeed, GC/MS untargeted metabolomics studies used similar conditions to ours to identify metabolites have reported that 78 metabolites were identified in serum [42] and liver [43], respectively.

In the plasma, seven of these 11 metabolic pathways were involved in lipid metabolism (fatty acid metabolism, fatty acid elongation in mitochondria, steroid biosynthesis, bile acid biosynthesis, glycerolipid metabolism, fatty acid biosynthesis, and phosphatidylinositol phosphate metabolism), three were involved in carbohydrate metabolism (inositol metabolism and inositol phosphate metabolism), and one was involved in amino acid metabolism (valine, leucine, and isoleucine degradation). In contrast, in the liver, seven of the nine metabolic pathways were involved in carbohydrate metabolism (amino sugar metabolism, glycolysis, fructose and mannose degradation, gluconeogenesis, the Warburg effect, the pentose phosphate pathway, and pyruvate metabolism), and the remaining two were involved in amino acid metabolism (glutamate metabolism) and nucleotide metabolism (pyrimidine metabolism).

Although the metabolites profiles of the plasma indicated seven metabolic pathways involved in lipid metabolism, neither of them was indicated in the liver. In this study, both plasma and liver tissue were extracted by methanol–chloroform–water, and the aqueous layer was used for GC/MS untargeted metabolomics. Although fatty acids are difficult to be distributed in the aqueous layer, they are often reported to be identified in plasma [44]. In agreement with this, we identified 9 fatty acids in the plasma, of which 4 fatty acids (i.e., nonanoic acid, myristic acid, palmitic acid, eicosanoic acid) were significantly increased. On the other hand, in the liver, only palmitic acid was identified, and there was no significant difference between the two groups. Although the reason why fatty acids are seldom identified in the liver remains unclear, such few fatty acids identified in the liver might indicate that no metabolic pathway is involved in lipid metabolism in the liver.

**Figure 3:**
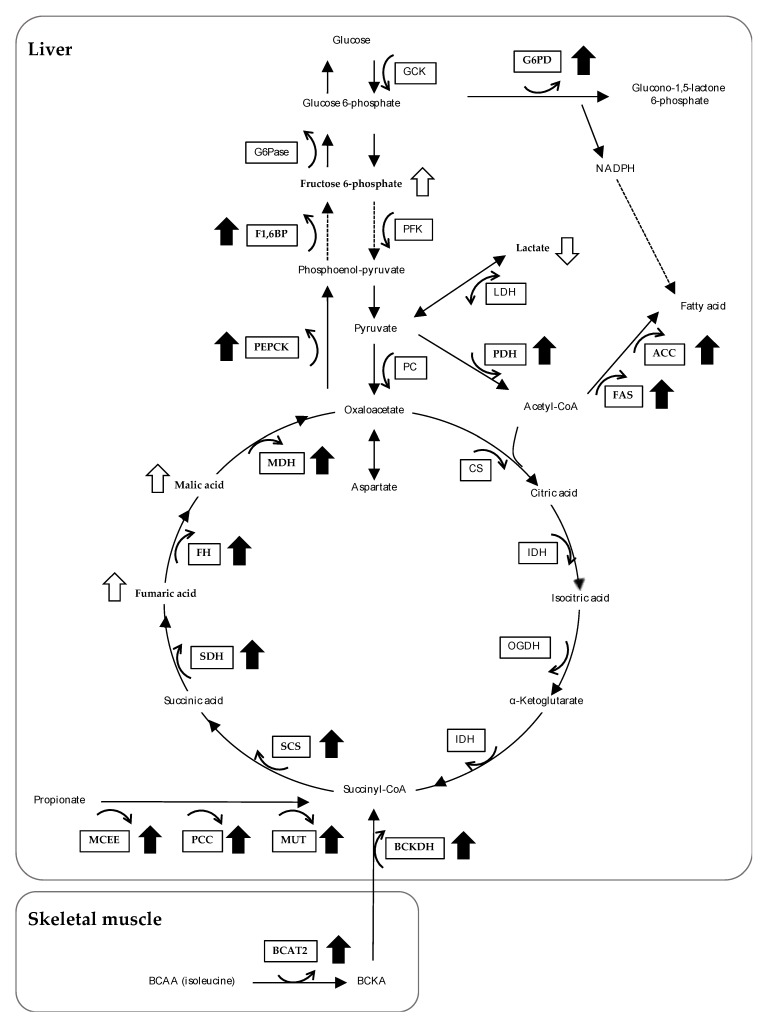
Integrated overview of the metabolic changes induced by feeding the OBFR diet. Open arrows indicate changes in the metabolites in the liver, and closed arrows indicate changes in gene expression in the liver. The metabolic scheme is based on information gathered from the KEGG PATHWAY Database (http://www.genome.jp/kegg/pathway.html). OBFR, outer bran fraction of rice; GCK, glukokinase; PFK, phosphofructokinase; PC, pyruvate carboxylase; PFH, pyruvate dehydrogenase complex; LDH, lactate dehydrogenase; G6PD, glucose-6-phosphate dehydrogenase; CS, citrate synthase; isocitrate dehydrogenase 3α; OGDH, oxoglutarate dehydrogenase; SCS, succinyl-CoA synthetase; SDH, succinate dehydrogenase B; FH, fumarate hydratase; MDH, Mmlate dehydrogenase; PEPCK, phosphoenolpyruvate carboxykinase; F1,6BP, fructose 1,6-bisphosphatase; G6Pase, glucose-6-phaosphatase; FAS, fatty acid synthase, ACC, acetyl-CoA carboxylase; MCEE, methylmalonyl CoA epimerase; PCC, propionyl-CoA carboxylase; MUT, methylmalonyl CoA mutase; BCAT, branched-chain aminotransferase; BCKDH, branched-chain α-ketoacid dehydrogenase; BCAA, branched-chain amino acid; BCKA, branched-chain α-keto acid, NADPH, nicotinamide adenine dinucleotide phosphate.

As mentioned above, because the liver plays a major role in metabolism and has a number of functions, including lipid metabolism, glucose metabolism, drug detoxification, and plasma protein synthesis, we explored the expression levels of genes encoding enzymes related to metabolic processes. The changes at the metabolites and gene expression levels induced by feeding the OBFR diet identified in this study are summarized in Figure 3.

Although there was no significant difference, feeding the OBFR diet tended to increase palmitic acid compared with their control diet counterparts (*p* = 0.09). In addition, the OBFR diet increased the mRNA expressions of SREBP1C, FAS, and ACC, suggesting that fatty acid synthesis may be enhanced. The OBFR diet also increased the mRNA expression of SREBP2, which is the master regulator of cholesterol synthesis and metabolism [45], whereas HMGR, the rate-limiting enzyme for cholesterol synthesis, did not differ between the two groups. Furthermore, neither CYP7A1, the rate-limiting enzyme for bile acid synthesis, nor CPT1a, the rate-limiting enzyme for fatty acid β-oxidation in the liver, were altered by the OBFR diet.

As the enrichment analysis by using the metabolites profiles of the liver indicated that changes in pyruvate metabolism and the pentose phosphate pathway had taken place, we examined the expression levels of genes involved in these processes in the rats’ livers. Feeding the OBFR diet increased the mRNA expression of PDH, but did not affect the mRNA expressions of PC, LDHa, or LDHb. In addition, the level of lactic acid was significantly lower in the rats fed the OBFR diet than in their control diet counterparts (Table 6). Furthermore, feeding the OBFR diet increased the mRNA expression of G6PD, which is a rate-limiting enzyme involved in the pentose phosphate pathway. These results suggest that feeding the OBFR diet may have enhanced either the conversion from pyruvate into acetyl-CoA, or NAPDH production, and consequently contributed to fatty acid synthesis by increasing substrate production in the livers of the rats.

The enrichment analysis by using the metabolites profiles of the liver also identified changes in either glycolysis or gluconeogenesis, and we therefore examined the expression levels of genes encoding rate-limiting enzymes for these two processes in the livers of rats. Feeding the OBFR diet did not affect the expression levels of the genes encoding GCK and PFK, whereas it significantly increased the expression levels of the genes encoding PEPCK mRNA and F1,6BP mRNA. In addition, G6Pase mRNA expression tended to be higher in the livers of the rats fed the OBFR diet. Furthermore, feeding the OBFR diet significantly increased the level of fructose 6-phosphate, which is an intermediate metabolite of gluconeogenesis, in the rats’ livers (Table 6). These results therefore suggest that feeding the OBFR diet may have enhanced hepatic gluconeogenesis than glycolysis. Our findings suggest that the OBFR diet-induced fatty acid synthesis and/or gluconeogenesis may be reasons that rats fed the OBFR diet could maintain their blood glucose and triacylglycerol levels, despite the fact that OBFR diet reduced the digestibility of ingested nutrients.

Certain non-carbohydrate organic compounds (e.g., glucogenic amino acids, pyruvate, lactic acid, and propionate) are known to be used as substrates for gluconeogenesis. In the liver, glucogenic amino acids are converted into pyruvate, succinyl-CoA, or fumaric acid. Feeding the OBFR diet decreased the levels of some glucogenic amino acids (tryptophan, isoleucine, and phenylalanine), and increased the fumaric acid and malic acid levels in the rats’ livers (Table 6). In addition, feeding the OBFR diet markedly increased the cecal propionic acid concentration. Propionic acid is found in the hepatic, portal, and peripheral blood [46,47], and is converted into succinyl-CoA. Furthermore, feeding the OBFR diet increased the expressions of the genes encoding MCEE, PCC, and MUT in the livers of the rats. These results suggest that either glucogenic amino acids or propionate may have been converted into pyruvate, succinyl-CoA, or fumaric acid in the livers of the rats fed the OBFR diet. Although feeding the OBFR diet did not change the mRNA expressions of CS, IDH, and OGDH, it significantly increased the mRNA expressions of SCS, SDH, FH, and MDH. These data support the hypothesis that feeding the OBFR diet increased the use of glucogenic amino acids and propionate as substrates for gluconeogenesis in the livers of the rats. Furthermore, in this study, since we found that feeding the OBFR diet markedly increased the concentrations of propionic acid in the intestinal contents, the increased propionic acid may have been converted to glucose via hepatic gluconeogenesis and contributed as an energy source in rats fed the OBFR diet.

Interestingly, the level of isoleucine in the cecal content was five-fold higher in the rats fed the OBFR diet than in the control rats (Table 4). In contrast, the levels of isoleucine in the liver and plasma were significantly lower in the rats fed the OBFR diet than in the control rats (Table 5 and Table 6). The enrichment analysis by using the metabolites profiles of the plasma identified a change in valine, leucine, and isoleucine degradation. Branched-chain amino acid (BCAA) is known to be metabolized solely in the liver [46,47], while it can be metabolized mainly in skeletal muscle by branched-chain aminotransferase (BCAT) to produce energy. We therefore examined the expression levels of the genes encoding BCAT2, BCKDCα, and BCKDCβ in the rats’ soleus muscles, and found that feeding the OBFR diet significantly increased the expression of all three genes (Figure S3A). These results suggest that isoleucine may have been metabolized in the skeletal muscles of the rats fed the OBFR diet. However, as feeding the OBFR diet did not change the expression levels of the genes encoding enzymes involved in glycolysis, β-oxidation, or the citric acid cycle in the soleus muscle (Figure S3B,C), the OBFR diet may not have affected the metabolic state of the skeletal muscle of the rats. It has been suggested that BCAA are converted into branched chain α-keto acids by BCAT in the skeletal muscle and that these branched chain α-keto acids are then metabolized by BCKDC and used as an energy source in the liver [47]. In this study, feeding the OBFR diet did not change the BCAT2 mRNA expression level in the liver, while it significantly increased the BCKDCα mRNA level (Figure 2G). These results support the hypothesis that feeding the OBFR diet enhanced isoleucine degradation in the skeletal muscle, and consequently contributed to energy production via branched chain α-keto acid metabolism in the livers of the rats.

However, the enrichment analysis by using the metabolites profiles of either the plasma or the liver also indicated that changes in the remaining metabolic pathways involved in carbohydrate metabolisms, amino acid metabolism, or nucleotide metabolism. One possible explanation for the reasons for changes in them might be partially due to changes in cecal metabolites. In cecal contents of the rats fed the OBFR diet, some sugar and sugar acid compounds (i.e., sucrose, mannitol, and glyceric acid) were increased compared with their control diet counterparts (Table 4), suggesting that these sugar and sugar acid compounds affects carbohydrate metabolisms either in liver or in the whole body of rats. In addition, although the enrichment analysis by using the metabolites profiles of the liver indicated that change in glutamate metabolism, it was a result from an increase in one compound (i.e., fructose 6-phosphate) in the liver. Therefore, it raised the possibility that cecal sugar metabolites might also affect fructose and mannose degradation, and consequently impact glutamate metabolism. Furthermore, the OBFR diet increased some nucleic acid metabolites (i.e., adenine and hypoxanthine) in rats’ cecal content (Table 4), suggesting that these metabolites might affect nucleotide metabolism. However, the reason why only pyrimidine metabolism was indicated in rats fed the OBFR diet, although the OBFR increased purine bases in rats’ cecal content, is unclear. Further studies are needed to gain insight into the effects of feeding the OBFR diet on nucleotide metabolisms in the liver and whole body of rats.

## 5. Conclusions

Eating OBFR reduced dietary digestibility and altered cecal metabolites (especially propionate and the other SCFAs) during the 21-day feeding period. In addition, hepatic metabolomics analysis indicated that alterations of pyruvate metabolism, the pentose phosphate pathway, and gluconeogenesis were accompanied by changes in the mRNA expressions of genes involved in these metabolic pathways. Furthermore, plasma metabolomics analysis indicated an alteration of BCAA degradation accompanied by increases in the mRNA expressions of gene encoding enzymes related to BCAA degradation in skeletal muscle and liver. Concordantly, these results suggest that the OBFR diet altered the concentrations of metabolites in the cecal contents, plasma, and liver, and the hepatic gene expressions of rats, and that this may have mainly contributed to carbohydrate metabolism in the liver.

## Figures and Tables

**Table 1:** Composition of experimental diets.

	Control Diet	OBFR ^1^ Diet
**Ingredients (g/100 g)**		
α-Cornstarch	52.95	50.97
Casein	20.00	19.27
Sucrose	10.00	10.00
Corn oil	7.00	6.09
Cellulose	5.00	3.62
Mineral mix (AIN-93G)^2^	3.50	3.50
Vitamin mix (AIN-93G)^3^	1.00	1.00
L-Cystine	0.30	0.30
Choline chloride	0.25	0.25
OBFR		5.00
**Analyzed contents ^4^**		
Moisture (g/100 g)	8.44	8.89
Crude protein (g/100 g)	17.38	17.20
Ether extract (g/100 g%)	6.09	7.06
Crude ash (g/100 g)	2.92	3.57
Nitrogen free extract (g/100 g)	63.22	60.77
Crude fiber (g/100 g)	1.95	2.52
Nutral detergent fiber (g/100 g)	6.67	6.90
Gross energy (Mcal/kg)	4.33	4.29

^1^ OBRF; outer bran fraction of rice bran. ^2^ Percent of the mineral premix: CaCO_3_ 35.7%, KH_2_2PO_4_ 19.6%, K_3_C_6_H_5_O_7_ H_2_O 7.078%, NaCl 7.4%, K_2_SO_4_ 4.66%, MgO 2.4%, FeC_6_H_5_O_7_ H_2_O 0.606%, 5ZnO 2CO_2_ H_2_O 0.165%, MnCO_3_ 0.03%, CuCO_3_Cu(OH)_2_ H_2_O 0.03% KIO_3_ 0.001%, Na_2_SeO_4_ 0.001025%, (NH_4_)6Mo_7_O_24_ 4H_2_O 0.000795%, Na_2_SiO_3_ 9H_2_O 0.145%, CrK(SO_4_)_2_ 12H_2_O 0.0275%, H_3_BO_3_ 0.00815%, NaF 0.00635%, NiCO_3_ 2Ni(OH_2_) 4H_2_O 0.00318%, LiCl 0.00174%, NH_4_VO_3_ 0.00066%, granulated sugar 22.1026%. ^3^ Percent of the vitamin premix: nicotinic acid 0.30%, Dl-calcium pantothenate 0.32%, vitamin B6 0.07%, vitamin B1 0.06%, vitamin B2 0.06%, folic acid, 0.02%, D-biotin (2%) 0.10%, vitamin B12 (0.1%) 0.25%, vitamin E (50%) 1.50%, vitamin A (500,000 IU/g) 0.08%, vitamin D3 (500,000 IU/g) 0.02%, vitamin K1 (phylloquinone) 0.0075%, granulated sugar 97.2125%. ^4^ Analyzed contents were measured in accordance with the Association of Official Agricultural Chemists procedures.

**Table 2:** Effects of feeding OBFR on growth performance and tissue weights of rats.

	Control			OBFR		
	(*n* = 7)			(*n* = 7)		
**Growth performance**						
Final body weight (g)	387.30	±	8.61	383.32	±	7.51
Body weight gain (g/21 days)	115.10	±	5.08	111.05	±	5.30
Food intake (g/21 days)	454.78	±	13.75	460.28	±	14.48
Food efficiency	0.25	±	0.01	0.24	±	0.01 *
**Tissue weight**						
Heart (g/100 g body weight)	0.28	±	0.01	0.28	±	0.01
Liver (g/100 g body weight)	3.38	±	0.14	3.53	±	0.15
Kidney (g/100 g body weight)	0.62	±	0.02	0.63	±	0.02
Abdominal fat (g/100 g body weight)	5.69	±	0.47	5.99	±	0.46
Soleus muscle (g/100 g body weight)	0.03	±	0.00	0.03	±	0.00
**Plasma**						
Glucose (mg/dL)	99.67	±	1.02	87.67	±	7.87
Triacylglycerol (mg /dL)	185.08	±	22.81	198.20	±	22.80
Total cholesterol (mg/dL)	103.17	±	8.20	98.33	±	11.66
3-Methylhisitidine (nmol/μL)	5.38	±	0.40	4.95	±	0.21

Values are means ± SEM (*n* = 7). OBFR; outer bran fraction of rice bran. * Significantly different from control group (*, *p* < 0.05) by Student’s *t*-test.

**Table 3:** Effects of feeding OBFR on organic acid concentration of rat’s cecal contents (nM/g feces).

	Control			OBFR		
	(*n* = 7)			(*n* = 7)		
Lactic acid	0.38	±	0.09	1.14	±	0.17 *
Acetic acid	39.75	±	2.01	147.67	±	6.97 *
Propionic acid	35.05	±	2.72	90.26	±	6.15 *
Isobutyric acid	7.20	±	0.28	7.12	±	0.52
Butyric acid	18.49	±	2.20	84.13	±	6.84 *
Isovaleric acid	5.67	±	0.31	8.65	±	0.43
Valeric acid	4.65	±	0.22	6.12	±	0.91
Total organic acid	105.61	±	4.23	345.09	±	14.55 *

Values are means ± SEM (*n* = 7). OBFR; outer bran fraction of rice bran. * Significantly different from control group (*, *p* < 0.05) by Student’s *t*-test.

**Table 4:** Effects of feeding OBFR on metabolites in cecal content of rats.

	Control			OBFR			*p*-Value
**Increased metabolites**							
Sucrose	100	±	22	166	±	17	0.049
3-Hydroxybenzoic acid	100	±	20	167	±	16	0.024
Glyceric acid	100	±	18	169	±	13	0.011
Glutaric acid	100	±	17	238	±	36	0.006
Mannitol	100	±	19	258	±	50	0.015
Sarcosine	100	±	11	267	±	64	0.033
Adenine	100	±	29	274	±	59	0.024
Hypoxanthine	100	±	34	511	±	173	0.048
Isoleucine	100	±	48	549	±	173	0.017
3-Hydroxyphenylacetic acid	100	±	33	737	±	124	0.001
**Decreased metabolites**							
Adenosine	100	±	19	10	±	0	0.002
Glycolic acid	100	±	32	17	±	3	0.042
Inosine	100	±	23	27	±	7	0.014
2-Aminoisobutyric acid	100	±	18	38	±	13	0.021
Proline	100	±	12	53	±	11	0.020
Valine	100	±	13	55	±	9	0.035
5-Aminovaleric acid	100	±	10	58	±	5	0.004
Thymine	100	±	7	59	±	6	0.001
Ornithine	100	±	12	64	±	10	0.048
Serine	100	±	9	65	±	6	0.011
Glycine	100	±	11	65	±	7	0.023
Pyroglutamic acid	100	±	8	75	±	6	0.030

Values are means ± SEM. OBFR; outer bran fraction of rice bran. Differences were considered significant at *p* < 0.05.

**Table 5:** Effects of feeding OBFR on metabolites in plasma of rats.

	Control			OBFR			*p* Value
**Increased metabolites**							
Mannose	100	±	17	465	±	59	0.006
Arabitol	100	±	4	179	±	22	0.009
Psicose	100	±	7	175	±	24	0.015
Sorbose	100	±	38	256	±	40	0.032
Sorbitol	100	±	11	254	±	65	0.047
Inositol	100	±	16	239	±	50	0.029
Nonanoic acid	100	±	22	234	±	50	0.039
Myristic acid	100	±	17	229	±	49	0.039
Palmitic acid	100	±	13	215	±	35	0.016
Eicosanoic acid	100	±	24	241	±	55	0.047
**Decreased metabolites**							
Succinic acid	100	±	13	25	±	16	0.011
β-Alanine	100	±	13	44	±	7	0.013
Isoleucine	100	±	31	22	±	7	0.040

Values are means ± SEM. OBFR; outer bran fraction of rice bran. Differences were considered significant at *p* < 0.05.

**Table 6:** Effects of feeding OBFR on metabolites in liver of rats.

	Control			OBFR			*p* Value
**Increased metabolites**							
Malic acid	100	±	7	134	±	8	0.009
Arabinose-5-phosphate	100	±	15	146	±	10	0.022
Ascorbic acid	100	±	41	229	±	30	0.027
N-Acetyl glucosamine	100	±	13	271	±	55	0.027
Uracil	100	±	31	183	±	15	0.030
Uridine	100	±	46	201	±	13	0.030
Glucono-1,5-lactone	100	±	35	316	±	67	0.030
Fumaric acid	100	±	10	135	±	11	0.036
Fructose 6-phosphate	100	±	25	216	±	44	0.040
Nonanoric acid	100	±	41	245	±	52	0.045
**Decreased metabolites**							
Gluconic acid	100	±	21	35	±	4	0.010
Lactic acid	100	±	35	15	±	13	0.020
Tryptophan	100	±	8	63	±	16	0.040
Isoleucine	100	±	9	77	±	9	0.040

Values are means ± SEM. OBFR; outer bran fraction of rice bran. Differences were considered significant at *p* < 0.05.

## Supplementary Materials

The following are available online at https://www.mdpi.com/2072-6643/12/2/430/s1, Figure S1: A heat map of the relative abundance of 64 metabolites that were detected in the cecal contents of rats; Figure S2: PCA score scatter plots obtained for the control and OBFR groups, of the metabolites in (A) the cecal content, (B) the plasma, and (C) the liver; Figure S3: Gene expression analysis of the soleus muscles from rats fed the OBFR diet; Table S1: The condition for peak detection and alignment of the GC/MS analysis data; Table S2: List of primers sequence used for quantitative real time polymerase chain reaction; Table S3, Effects of feeding OBFR on feed digestibility of rats (%).
