# Supplementary material for: Feeding the Outer Bran Fraction of Rice Alters Hepatic Carbohydrate Metabolism in Rats
Source: Nutrients. 2020 Feb 7;12(2):430. doi: 10.3390/nu12020430 (PMC7071268; doi:10.3390/nu12020430)
Supplement: Supplementary file 1 [file nutrients-12-00430-s001.pdf]

Supplementary Materials:

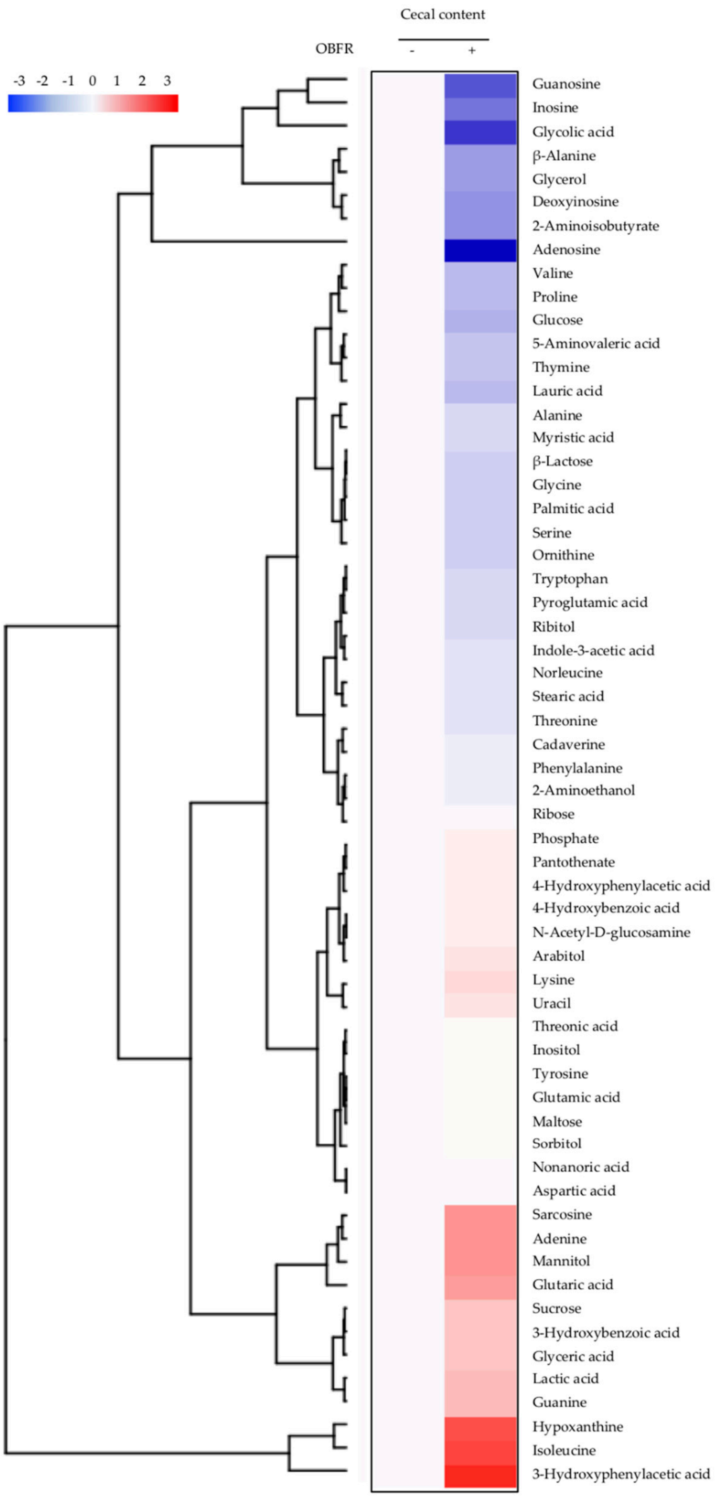

Figure S1. A heat map of the relative abundance of 64 metabolites that were detected in the cecal contents of rats.

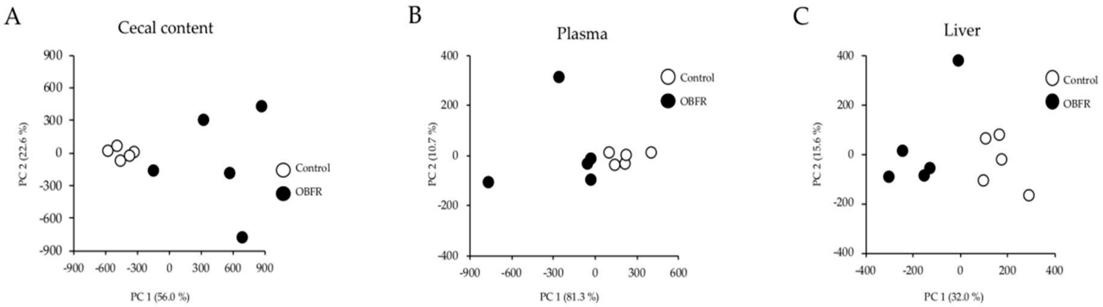

Figure S2. PCA score scatter plots obtained for the control and OBFR groups, of the metabolites in (**A**) the cecal content, (**B**) the plasma, and (**C**) the liver.

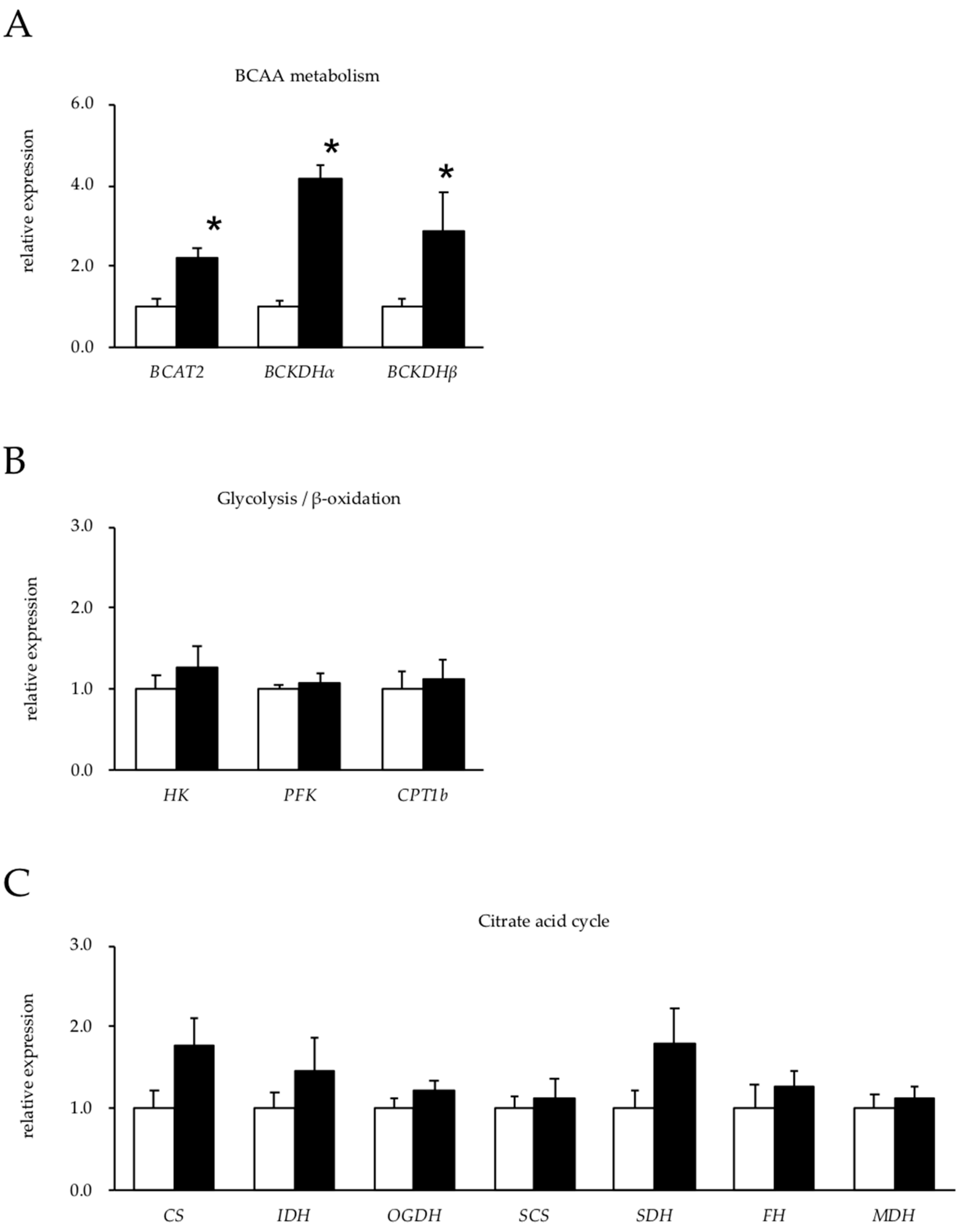

Figure S3. Gene expression analysis of the soleus muscles from rats fed the OBFR diet

Table S1. The condition for peak detection and alignment of the GC/MS analysis data

**Data type**	
Data type	Centroid
Ion mode	Positive
Accuracy type	IsNominal
Data collection parameters	
Mass range begin	0
Mass range end	1000
Retention time begin	0

Retention time end	100
**Peak detection parameters**	
Smoothing method	Liner weighted moving average
Smoothing level	2
Average peak height	20
Minimum peak height	2000
Mass slice width	0.5
Mass accuracy	0.5
**MS 1 Dec parameters**	
Sigma window value	1
Amplitude cut off	200
**Identification**	
MSP file	GCMS DB_InertCap 5MS-NP_GLscience.msp
Retention type	RI
RI compound	Alkanes
Retention time tolerance	0.5
Retention index tolerance	20
EI similarity library tolerance	70
Identification score cutt off	70
**Alignment parameters setting**	
Retention time tolerance	RT
Retention index tolerance	20
Retention time tolerance	0.075
EI similarity tolerance	70
Retention time factor	0.5
EI similarity factor	0.5
Peak count filter	0
QC at least filter	TRUE

Table S2. List of primers sequence used for quantitative real time polymerase chain reaction.

Gene	abbreviation		Sequence (5'-3')
Sterol regulatory element-binding protein 1C	SREBP1c	Forward	GCC CAC AAT GCC ATT GAG A
		Reverse	CAG GTC CTT GAG CTC CAC AAT C
Sterol regulatory element-binding protein 2	SREBP2	Forward	GCC TAG CCC TCG TGT CTT GTG TA
		Reverse	CCA GCA CAT GGT GCA TGT TTC
Fatty acid synthase	FAS	Forward	AGC ATA TCC CTGGAA ACA GGT GAC
		Reverse	TCT GTG GAT AGG ACT GAA TGC TGT G
Acetyl-CoA carboxylase	ACC	Forward	ACC AGC CTC TGT CAG CTC AG
		Reverse	CCC CCA AAA CGA GTA ACA AA
Hydroxymethylglutaryl-CoA reductase	HMGR	Forward	AGA CCT CGC TCA GGA GCA TTG
		Reverse	GCC ATT CAT GTG GCT CCA TC
Cholesterol 7 alpha-hydroxylase	CYP7A1	Forward	GCT GCA GCG AGC TTT ATC CAC
		Reverse	CCA GTC AGC CTG GGT TGC TA
Carnitine / palmitoyl-transferase 1	CPT1a	Forward	GAG CAA TAG GTC CCC ACT CAA
		Reverse	ATC TGT TTG AGG GCT TCG TGG
Pyruvate dehydrogenase complex,	PDH	Forward	CTC ACG CCT ATG CTA CTG CT
		Reverse	AGA TGC TTT GGG CCT TCT CC
Pyruvate parboxylase	PC	Forward	CTC TGG AAG GCG ATG ACC TC
		Reverse	CCA TGG GAC AGG ACC TCA AG
Lactate dehydrogenase A	LDHa	Forward	TGC ACT AAG CGG TCC CAA A
		Reverse	AAC AGC ACC AAC CCC AAC AA
Lactate dehydrogenase B	LDHb	Forward	TTG TCT GGA CAA GAT GGC AAC
		Reverse	ATC TTG TTG TTC GGG ACG GC
Glucose-6-phosphate dehydrogenase	G6PD	Forward	TGT GGA GAA TGA ACG GTG GG
		Reverse	CGC GGA ACT GAA GTC TCA CT
Glucokinase	GCK	Forward	ACT GCC GAG ATG ATG AAG CA
		Reverse	AGC CCT TGG TCC AAT TGA GG
Phosphofructokinase	PFK	Forward	GGT GAC GCG CAA GGT ATG AA
		Reverse	GCC CTC GTA GCC CTC GTA GA
Phosphoenolpyruvate carboxykinase	PEPCK	Forward	GGA TGT GGC CAG GAT CGA AA
		Reverse	ATA CAT GGT GCG GCC TTT CA
Fructose 1,6-bisphosphatase	F1, 6BP	Forward	TTA CGT CAT GGA GAA GGC CG
		Reverse	TCT CAG TGG GGA CGA TGT CT
Glucose-6-phaosphatase	G6Pase	Forward	GCG TGC CAT AGG ACT CAT CA

		Reverse	CAC CAG CAA ACAATT GCC CA
Methylmalonyl CoA epimerase	MCEE	Forward	GGT ATT GGG GCA CTG CCT TC
		Reverse	CTA CAA GCT TGC ACA GCC AC
Propionyl-CoA carboxylase	PCC	Forward	TTC AAG CCA TGG GTG ACA AGA
		Reverse	CCT TGC AAT TCT GAC GGC TT
Methylmalonyl CoA mutase	MUT	Forward	GAA GAA CTG CCG GGA GTG AA
		Reverse	CCA CAG TAC TAA AGC CCG CA
Citrate synthase	CS	Forward	CTC TGC ATG GAC TAG CAA ACC
		Reverse	CAT CTG ACA CGT CTT TGC CG
Isocitrate dehydrogenase 3α	IDH	Forward	CTG TGC ACA AGG CCA ACA TC
		Reverse	AGA ACA TCG AAC TGG GAC GG
Oxoglutarate dehydrogenase	OGDH	Forward	TCT CTC ATC AGG GCG TAT CAG
		Reverse	GCC ATA GAA CCC AAG TTT GTC TGT
Succinyl-CoA synthetase	SCS	Forward	ATA CGG CAC GGG TCT TAC AC
		Reverse	CCT GCT GGC TGT GAA AGG TA
Succinate dehydrogenase B	SDH	Forward	CGA CCT ACA AGG AGA AGC GG
		Reverse	TTG AAG GGA CTC ACG CCA GA
Fumarate hydratase	FH	Forward	ATT CGC TTC CTC GGT TCT GG
		Reverse	AAT GTC CAT TGC TGC CTC CA
Malate dehydrogenase 2	MDH	Forward	GAC CCA GCT CGA GTC AAT GT
		Reverse	GGA AAG TCA ACC TTG GGG GT
Branched-chain aminotransferase	BCAT	Forward	CCA GAA CTG CTG GAG TGC AT
		Reverse	TGG AGT CTC CGG GGA AGT AG
Branched-chain α-ketoacid dehydrogenase complex alpha	BCKDHα	Forward	ATC TCC GGC ATC CCC ATC TA
		Reverse	CCT CCT CGC CAT AGT TGG TC
Branched-chain α-ketoacid dehydrogenase complex beta	BCKDHβ	Forward	CGG AAA TCC AGT TTG CCG AC
		Reverse	GTG AGG CTC CCA CAG TTG AA
Hexokinase	HK	Forward	TCT TTT GTC CGG AGC ATC CC
		Reverse	ACC CGG AAA TTC GTT CCT CC
Ribosomal protein S18	RPS18	Forward	AAG TTT CAG CAC ATC CTG CGA GTA
		Reverse	TTG GTG AGG TCA ATG TCT GCT TTC

Table S3. Effects of feeding OBFR on feed digestibility of rats (%).

	Control (n = 7)			OBFR (n = 7)		
Crude protein	94.10	±	0.24	92.84	±	0.49 *
Ether extract	92.12	±	0.45	90.19	±	0.74 *
Crude fiber	31.79	±	4.66	30.60	±	6.56
Crude ash	57.37	±	1.58	53.04	±	0.95
Nitrogen free extracts	94.69	±	0.13	93.90	±	0.40 *
Neutral Detergent Fiber	14.48	±	8.13	32.96	±	6.34
Gross energy	92.57	±	0.21	90.40	±	0.17 *

Values are means ± SEM (n = 7). OBFR; outer bran fraction of rice bran. * Significantly different from control group (*, $P < 0.05$) by student's T-test.
